# Gender differences in human single neuron responses to male emotional faces

**DOI:** 10.3389/fnhum.2015.00499

**Published:** 2015-09-14

**Authors:** Morgan Newhoff, David M. Treiman, Kris A. Smith, Peter N. Steinmetz

**Affiliations:** ^1^Department of Neurology, Barrow Neurological InstitutePhoenix, AZ, USA; ^2^Department of Neurosurgery, Barrow Neurological InstitutePhoenix, AZ, USA

**Keywords:** gender differences, human single neuron, amygdala, emotional expression, facial experssion

## Abstract

Well-documented differences in the psychology and behavior of men and women have spurred extensive exploration of gender's role within the brain, particularly regarding emotional processing. While neuroanatomical studies clearly show differences between the sexes, the functional effects of these differences are less understood. Neuroimaging studies have shown inconsistent locations and magnitudes of gender differences in brain hemodynamic responses to emotion. To better understand the neurophysiology of these gender differences, we analyzed recordings of single neuron activity in the human brain as subjects of both genders viewed emotional expressions. This study included recordings of single-neuron activity of 14 (6 male) epileptic patients in four brain areas: amygdala (236 neurons), hippocampus (*n* = 270), anterior cingulate cortex (*n* = 256), and ventromedial prefrontal cortex (*n* = 174). Neural activity was recorded while participants viewed a series of avatar male faces portraying positive, negative or neutral expressions. Significant gender differences were found in the left amygdala, where 23% (*n* = 15∕66) of neurons in men were significantly affected by facial emotion, vs. 8% (*n* = 6∕76) of neurons in women. A Fisher's exact test comparing the two ratios found a highly significant difference between the two (*p* < 0.01). These results show specific differences between genders at the single-neuron level in the human amygdala. These differences may reflect gender-based distinctions in evolved capacities for emotional processing and also demonstrate the importance of including subject gender as an independent factor in future studies of emotional processing by single neurons in the human amygdala.

## Introduction

Since gender is such an integral part of human identity, gender differences in the human brain have long been a source of interest and dissent amongst researchers. Anatomically, there are well-documented gender differences in relative volumes of neural structures including the frontal cortices, the hypothalamus, and the amygdala (Goldstein et al., 2001). While researchers have offered evidence of the biochemical processes which cause these anatomical differences to arise (Nugent and McCarthy, 2011; Uddin et al., 2013), it is less understood how these differences are translated into a difference in emotional processing or behavior.

In total, more than two thousand neuroimaging studies exploring emotional processing have been published since 1990. While many have reported significant gender differences in hemodynamic changes, the location, extent and direction of these differences have varied widely, leading several groups of researchers to search for consistent patterns by performing meta-analyses. The results of these analyses have been mixed, particularly with regards to the present study's region of interest: the amygdala.

The most recent meta-analysis, by Stevens and Hamann (2012), evaluated studies whose stimuli evoked emotions broadly classified as “positive” (amusing, pleasant or erotic) or “negative” (anger, fear, disgust, sadness) and evaluated results in terms of a positive vs. negative valence. The authors found that men were significantly more often reported as having higher responses to positive stimuli in the left amygdala (amongst other structures), while women were reported as being more responsive to negative stimuli in the same region. By contrast, Fusar-Poli et al. (2009) performed a meta-analysis of 105 studies in which the stimuli were limited to emotional faces, and found that these stimuli significantly increased activity in the right amygdalae of men compared to women, with no significant effect of gender in the left amygdala. A prior meta-analysis by Sergerie et al. (2008) of emotional processing imaging studies reporting amygdala activation did not find gender to be a predictive variable for lateralization of activity. While the contradictory results of these meta-analyses may have been partly due to variations in study inclusion criteria and statistical methods, their inconsistencies make it difficult to predict what types of differences in single neuron firing would be expected between male and female participants.

To clarify the effects of gender on human neural response to emotional faces, we examined single neuron firing recorded in the human brain during an experiment showing faces which incidentally varied emotional expression. While this experiment was designed to vary the race of synthetic faces, the faces also depicted positive, neutral, and negative expressions. This allowed us to directly measure differences in the response of single neurons to emotional expressions between the male and female participants. We recorded from four clinically mandated brain areas, including the amydala, a structure proven in lesion studies to be essential for correctly perceiving the emotions of others (Adolphs et al., 1994; Becker et al., 2012), and which has been shown to contain neurons significant for facial emotional processing in single-unit recording experiments in monkeys (Kuraoka and Nakamura, 2007). Since we recorded from both hemispheres, we were also able to determine whether neural activity was lateralized.

In brief, we found a significant gender difference in single-neuron firing rates in response to emotional faces. This difference was localized to the left amygdala, where a greater number of neurons in men fired significantly in response to stimuli than neurons in the same location in women. The men and women in the study also differed behaviorally in response time and accuracy in an emotion identification task.

## Methods

### Participants

Single-neuron firing activity was recorded from microwires implanted in 14 pharmaco-resistant epilepsy patients, 6 male and 8 female (13 right handed, ages 21–56, mean age = 29) at the Barrow Neurological Institute. Table 1 shows the age and characteristics of illness for each subject enrolled in this study. Patients were being evaluated for possible resection of an epileptogenic focus. Each patient receiving depth electrode monitoring was asked to participate in the study and so no attempt was made to balance the gender of the patients enrolled. Data were recorded from clinically mandated brain areas including the amygdala, the hippocampus, the anterior cingulated cortex and the prefrontal cortex. All patients granted consent to participate in the experiment using a protocol approved by the Institutional Review Board of Saint Joseph's Hospital and Medical Center.

**Table 1:** **Subject characteristics and clinically identified seizure foci**.

**Age**	**Gender**	**Handedness**	**Brain areas with Foci**
38	Male	Right	Right temporal lobe
54	Female	Right	Left amygdala, left hippocampus, right hippocampus
42	Male	Right	Left amygdala, left hippocampus
35	Male	Left	Right amygdala, right hippocampus
41	Male	Right	Left hippocampus, left frontal lobe
21	Female	Right	Left amygdala, left hippocampus
24	Female	Right	Right amygdala, right hippocampus, right frontal lobe
23	Male	Right	Right amygdala, right hippocampus
54	Female	Right	Left hippocampus
56	Female	Right	Right occipetal lobe
40	Female	Right	Right amygdala, right hippocampus
54	Female	Right	Right hippocampus
20	Male	Right	Right temporal lobe
53	Female	Right	Right amygdala, right hippocampus, left amygdala, left hippocampus

### Experimental procedures

**Figure 1:**
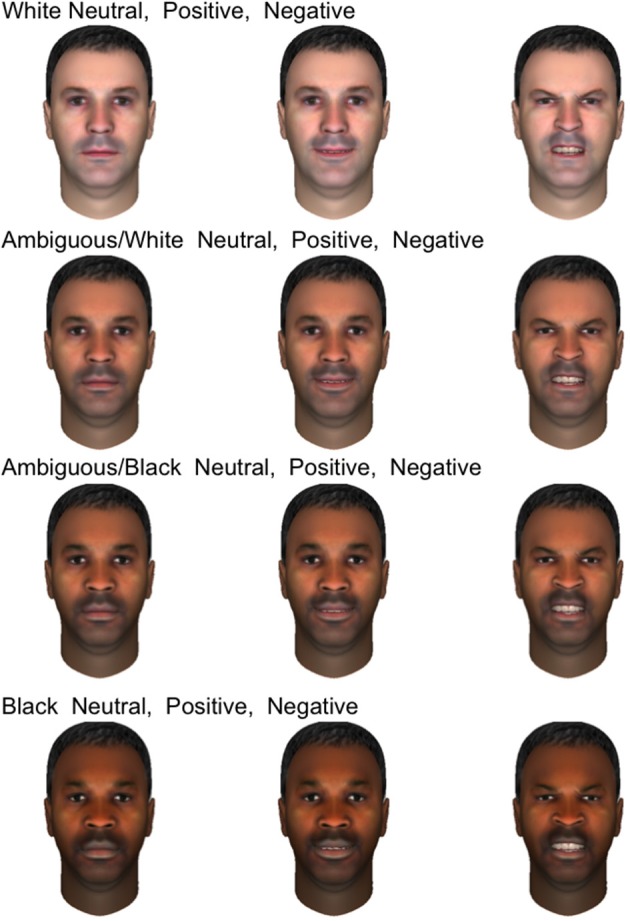
**Examples of emotional stimuli presented to subjects**. The faces shown are generated from a single prototype face image and depict four races, from top to bottom rows: white, ambiguous white, ambiguous black, and black. In each row, three emotional expressions are shown: neutral, positive, and negative.

Participants were presented with a set of 120 synthetic male faces which were originally designed to test the effects of race on the firing of human single neurons while also displaying varying emotional expressions. These faces were created using FaceGen Modeler (Inversions, 2006) to permit smooth variation between prototypical Caucasian and African-American. Because these synthetic faces contained variation in emotional expression as well as race, we were able to examine how neural responses to both the displayed emotion and race of the face depended on the gender of the participant. Only male faces were shown in the original design to minimize additional independent variation and maximize power to distinguish between races. Given the adventitious design of this analysis, we did not show both male and female faces. Figure 1 displays 12 of the emotional faces utilized in the present study which were generated from a single prototype face image. Ten prototype face images were used to generate the entire set. All three expressions appeared in equal numbers throughout the experiment (40 of each, presented in random order), and the emotions depicted were validated in two perceptual experiments with undergraduate volunteers from Arizona State University (see SI Methods). Stimulus race had a significant effect on firing rate (will be reported separately), but men and women did not differ with regards to this response, making stimulus race of lesser importance to the present study.

**Figure 2:**
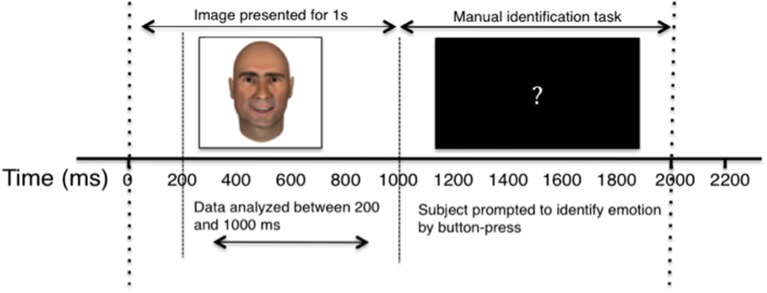
**Diagram of single experimental trial**. The horizontal axis depicts the passage of time, in milliseconds. Dashed lines separate key stages of the experiment, and labeled arrow bars distinguish between the recording and behavioral segments.

Participants were presented with centrally-located facial images appearing on a laptop screen subtending approximately 11° visual angle. Images appeared for 1000 ms, and were followed by a black screen with a centrally-located white question mark for 2000 ms, during which time the participants were instructed to classify the emotion of the preceding face. Participants identified emotion by pressing one of three buttons on a trackpad labeled “Sad,” “Happy,” and “Neutral” and were instructed to use the “Sad” button for any sad, angry, or negative emotion. Each face in the study was presented six times, with each experiment consisting of 720 trials in total. For a diagrammatic representation of the behavioral task, see Figure 2.

Cells were recorded continuously, but only firing between 200 ms and 1000 ms after image presentation (prior to manual identification task) was included in the analysis. Trials in which participants responded early, pressed more than one identifying key, or failed to respond were eliminated from analyses. The elimination of error trials did not affect the reported results.

### Microwire implantation, signal amplification, and spike sorting

We used surgical and recording methods which we have previously described in Valdez et al. (2013). In brief, nine microwires were implanted stereotactically (Medtronic StealthStation) with a 1.5T structural MRI through skull bolts at each recording site protruding from the clinical depth electrodes used to locate epileptogenic focus (Dymond et al., 1972; Fried et al., 1999). In the hippocampus, the target for microwire tip placement was the mid-body of the hippocampus. In the amygdala, the target was the center of the amygdala; in the anterior cingulate cortex, the target was the anterior cingulate gyrus, above and behind the genu of the corpus callosum; in the ventromedial prefrontal cortex, the target was just below the anterior cingulate gyrus and corpus callosum, in the most anterior portion of the gyrus rectus. Using these techniques, the error in tip placement is estimated to be ±2 mm (Mehta et al., 2005). While this resolution is insufficient to determine subfields within the hippocampus or nuclei within the amygdala, it is sufficient to allow discovery of neural firing differences between major brain areas and sides of the brain.

**Figure 3:**
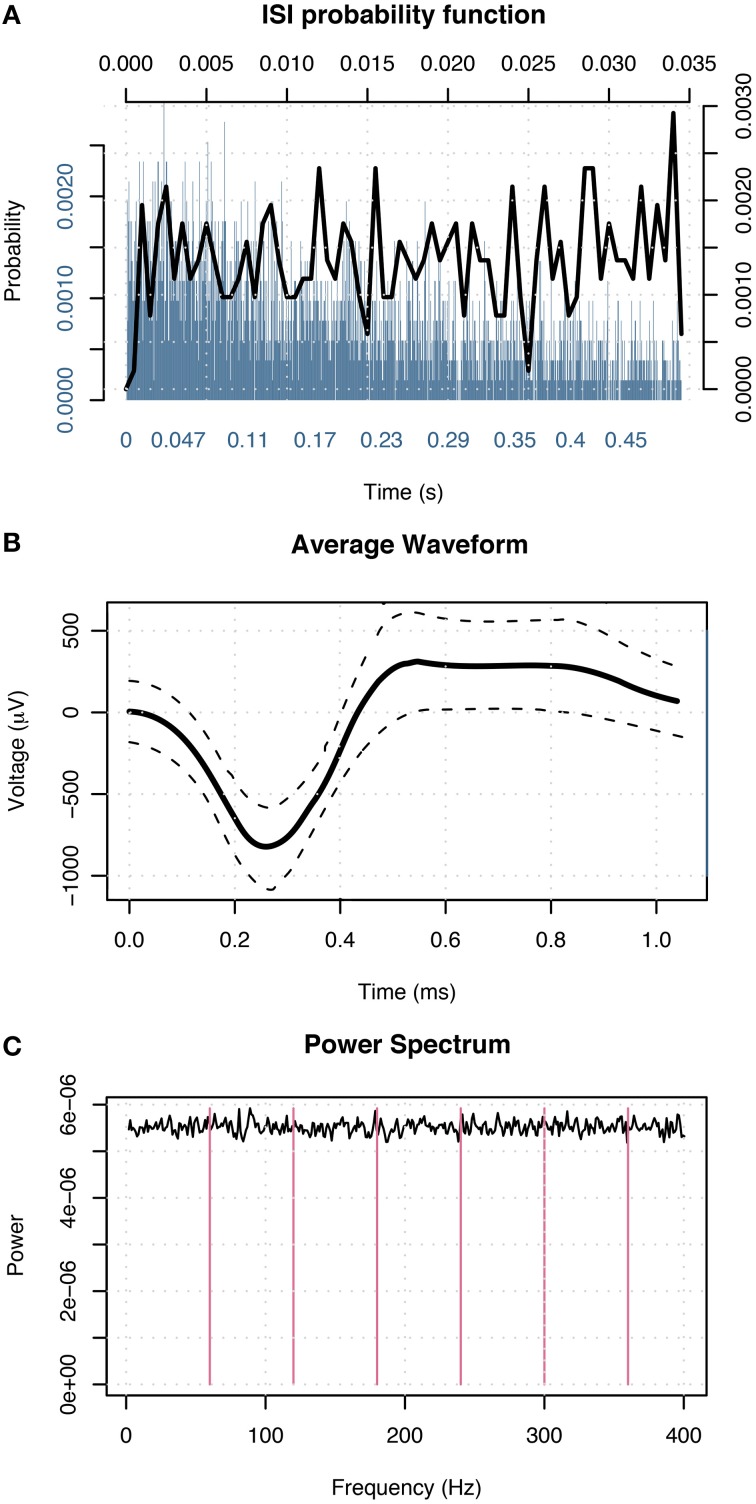
**Events in a cluster in the left amygdala of a male classified as single-unit activity. (A)** Distribution of interspike intervals over two duration scales: broad range 0–0.5 s (blue) and narrow range 0–0.035 s (black) shown on x-axis. The y-axis designates probability of interval. **(B)** Average waveform shape. Time (ms) is shown on the x-axis. The y-axis denotes scale in μV. The dashed lines indicate ± 1 SD at each sample point. **(C)** Power spectral density of event times. The x-axis displays frequency in Hz; magenta lines indicate primary and harmonics of the power line frequency (60 Hz). The y-axis shows power spectral density in events 2/Hz.

Following patients' recovery from surgery, the microwires were connected to headstage amplifiers which applied a 400x gain to yield eight recording channels. Microwire tips continuously recorded extracellular action potentials corresponding to single-neuron activity. Possible action potentials were high-pass filtered to determine event shape, and all events recorded from individual channels were grouped into sets of similar waveform shape (clusters) with the open-source clustering program KlustaKwik (http://klustakwik.sourceforge.net). Post-sorting, each cluster was classified as either noise, multi-unit or single-unit activity per the criteria listed in Valdez et al. (2013) Table 1. Figure 3 illustrates events in a cluster classified as single-unit activity after sorting.

This spike-sorting technique has been previously used in multiple publications (Steinmetz et al., 2013; Valdez et al., 2013, 2015; Wixted et al., 2014). In our experience, this technique (Valdez et al., 2013) produces results comparable to prior reports in other laboratories (Viskontas et al., 2007) in terms of recorded waveform shapes, inter-spike intervals, and firing rates (also see Wild et al., 2012 regarding variability in spike sorting depending on the particular waveforms shapes being detected). While it is important to note that these and other reports of human single-unit recordings (Kreiman et al., 2000; Steinmetz, 2009) do not achieve the quality of unit separation achievable in animal recordings (Hill et al., 2011), they nonetheless represent neural activity at a much finer spatial and temporal scale than otherwise achievable.

## Analysis

Each neuron in the study was classified according to brain area, side, recording quality, and gender of participant. Only neurons with well-isolated single unit activity were included in analyses. Our analysis here parallels that we recently used to examine object encoding (Valdez et al., 2015). We created a nested set of generalized linear models (McCullagh and Nelder, 1989) in R (R Development Core Team, 2009) testing firing rate as a function of stimulus affect for each neuron in each experiment (*n* = 936). Image luminance and contrast were included as additional factors given their recently demonstrated effect on firing rate in the amygdala (Steinmetz et al., 2011). Model 1 contained only a constant. Model 2 contained a constant plus the addition of luminance and contrast factors. Model 3 contained constant, luminance and contrast factors plus the addition of the stimulus emotion. Models 2 and 3 were compared with an ANOVA *F*-test (*df* = 2) for each neuron in the study to determine whether the addition of the affect stimuli factor improved goodness of fit (McCullagh and Nelder, 1989). Neurons with a resultant *p*-value < 0.05 were deemed significantly affected by stimulus emotion.

Binomial tests were used to determine if firing activity within brain areas was significantly affected by differences in stimuli facial emotion. Using a binomial distribution, we tested the probability of the observed outcome plus all less likely outcomes against the outcome expected by random chance (Stuart et al., 1999). Our expected outcome was that 5% of neurons in each area were significant for stimuli affect and the *p*-values for this test were adjusted using a Benjamini–Hochberg (BH, Benjamini and Hochberg, 1995) adjustment for false discovery rate.

To determine whether a sex difference was present within a tested brain area, neurons were classified by the gender of the subject. A Fisher's Exact Test (Fisher, 1922) was conducted to determine whether the ratios of neurons with a significant or non-significant response in each brain area in men and women differed significantly, based on a null hypothesis that these ratios are equal. The *p*-values for these tests were also corrected using a BH correction (Benjamini and Hochberg, 1995).

To ensure this, gender-based trends did not result from the presence of a small number of data outliers, we examined the distribution of *F*-test *p*-values for both genders in addition to ratios of significant and non-significant neurons. We plotted the quantiles of the probability distributions of *p*-values < 0.2 for neurons in men against those in women in Quantile-Quantile (Q-Q) plots (Chambers et al., 1983). Equal distributions generate points along the line y = x. Deviation from this 45° line indicates that the distributions differ with regards to dispersal. Since we plotted *p*-values of neurons in males along the y-axis and those from females along the x-axis, points deviating rightward from the central line indicate greater dispersal of values in females than in males.

While prior studies have often restricted analysis of the effects of independent factors, such as emotion, to neurons with responses that differ from background firing, we do not do so, because this form of pre-selection can lead to erroneous conclusions (Steinmetz and Thorp, 2013). Instead, we applied multinomial logistic regression (MLR McCullagh and Nelder, 1989) to determine which categories of facial emotion yielded firing rates which differed most significantly from background firing (essentially a simplified version of the point-process framework proposed by Truccolo et al., 2005). This technique provides a means of examining the relative effects of multiple explanatory covariates on a nominal dependent variable by constructing linear predictor functions for each covariate. For our purposes, we created a model for each cluster to predict the affect category of the image shown given the neuron's firing rate. Examination of the model coefficient for each category indicates the likelihood of obtaining observed firing data in each category assuming it was background firing. Statistically reliable changes in coefficient values from zero were determined using multivariate *t*-tests (Hosmer and Lemeshow, 2000, Chap. 2), one for each neuron. Once again, neurons were classified by brain area, side, and gender of participant. Fisher's Exact Tests were applied to the aforementioned tables to determine whether males and females differed significantly in response to the different categories of facial emotion.

## Results

**Figure 4:**
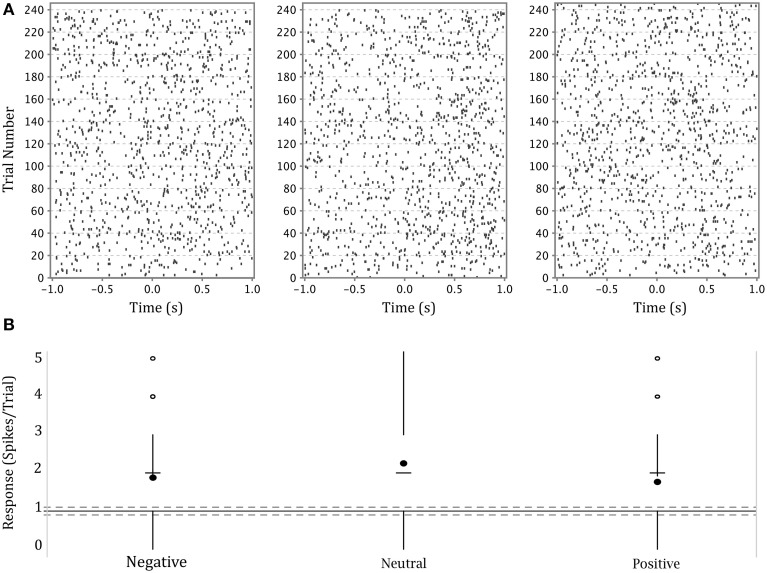
**(A)** Upper panel: raster plots of the responses of a single neuron in the left amygdala of a male participant to each of three categories of affect. Y axis—trial numbers. The points on each line represent the firing of action potentials during each trial. X axis—time in seconds from onset of stimulus. The rasters are specific to the affect categories labeled at the base of part B. **(B)** Lower panel: Modified box-plot of the firing rate of the same neuron in the left amygdala of a male participant as a function of stimulus affect. The center dots show mean response per affect category. The horizontal dashes show median response per affect category. Vertical lines extend from $\frac{\pm \left(1.58*IQR\right)}{\sqrt{\left(n\right)}}$ (where *IQR* = interquartile range, *n* = number of observations; equivalent to a 95% confidence interval for differences between medians, Chambers et al., 1983, p. 62) to the data point furthest from the median which is no more than ±(1.5^*^IQR) beyond the first or third quartiles. Open circles show responses outside that range. Solid gray line shows the mean of background firing; dashed gray lines at $\frac{\pm \left(1.58*IQR\right)}{\sqrt{\left(\hat{n}\right)}}$ of the background firing ($\hat{n}=\text{mean number of presentations of an object}$), representing a 95% confidence interval for the median of background firing.

The firing activity of a significant number of neurons depended on stimulus affect. Figure 4 shows an example of a neuron in the left amygdala of a male subject with a significant effect of emotion (This is the same neuron whose waveform is shown in Figure 3). The higher density of dots in each raster plot following presentation of the stimulus demonstrates a generalized heightened firing in response to the presentation of any emotional faces as compared to the preceding black screen. The neuron represented in this figure showed a stronger response to neutral expressions (center panel), particularly between 500 and 1000 ms after stimulus presentation. This is also apparent in the modified box plot in the lower panel where the mean of firing for this emotion is above that for other emotions. (Note that we show a modified box-plot in order to visually compare responses to firing in all background intervals, rather than simply comparing to background intervals preceding trials depicting a particular emotion.)

To compare the activity of neurons in populations across several brain areas, we tested for a selective response to emotion in all neurons in all brain areas. (We do not limit analysis to only neurons with a generalized visual response to avoid the errors which can arise from such pre-selection, Steinmetz and Thorp, 2013). Table 2 shows the number of neurons in each brain area with firing rates significantly affected by stimulus emotion. The rightmost column lists the *p*-values of a binomial test of whether the fractions of neurons with a significant response to emotion are greater than that expected by chance. The amygdala was the only brain area in which the firing rates of a significant number of neurons were influenced by stimulus emotion.

**Table 2:** **Neurons significant for emotion by brain area**.

**Area**	**Total # neurons**	**# Significant**	**Binomial *p*-value**	**Adjusted *p*-value**
A	236	23	0.0024	0.0096
AC	270	20	0.091	0.18
H	256	11	0.77	0.86
PF	174	9	0.86	0.86

Within the amygdala, we found this activity was highly lateralized, and differed measurably between men and women. Tables 3, 4 show the numbers of neurons with a significant response split by gender in the left and right amygdala, respectively. In the left amygdala, 15% of neurons had a significant effect of affect. When amygdalar neurons were split by gender of participant, a clear difference emerged. In the left amygdala of males, 23% of neurons had a significant effect of affect, whereas only 7.9% of neurons in females had such an effect. Binomial tests found this fraction to be significant in males (*p* = 7.2E-07) but not in females (*p* = 0.28). A Fisher's Exact Test comparing these two ratios found the ratios to be significantly different between males and females (*p* = 0.013).

**Table 3:** **Neurons significant for emotion in left amygdala**.

**Gender**	**Total # neurons**	**# Significant**	**Binomial *p*-value**	**Adjusted *p*-value**
Female	76	6	0.28	0.56
Male	66	15	7.2 x 10^−7^	2.9 x 10^−6^

**Table 4:** **Neurons significant for emotion in right amygdala**.

**Gender**	**Total # neurons**	**# Significant**	**Binomial *p*-value**	**Adjusted *p*-value**
Female	54	1	0.52	0.70
Male	40	1	0.72	0.72

**Figure 5:**
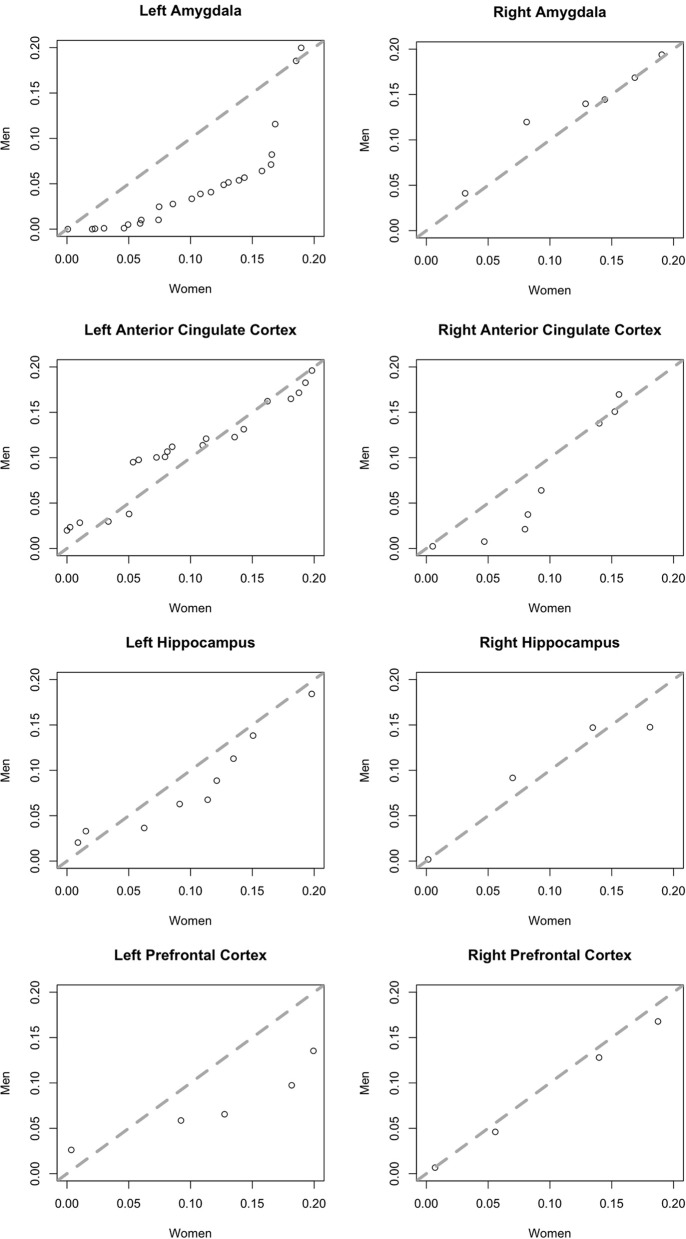
**Quantile-quantile plots of the distributions of *p*-values of an F-test for an effect of affect on neurons in men (y-axis) plotted against those in women (x-axis)**. Plots include more significant values, *p* < 0.2. The central diagonal dashed line shows the expected result if the distributions in men and women were the same.

To better illustrate differences between brain areas in the fractions of neurons with a significant effect of affect, we generated quantile-quantile (Q-Q) plots of the *p*-value distributions of tests of the effect of affect in male vs. female subjects. The distribution in the left amygdala (Figure 5) demonstrates that the significant difference we found in this area between men and women is not due to the presence of a small number of potential outliers, but rather an overall shift in the distribution. This plot contrasts sharply with the plots of brain areas in which no significant gender difference was found (Figure 5), and in which the data points fall close to the central diagonal line. The only other brain area in which the Q-Q plot of *p*-value distributions revealed a potential trend is the right amygdala (Figure 5), though this difference failed to reach significance.

We next determined which categories of emotion (negative, neutral, or positive) particular neurons were responding to using multinomial logistic regression (MLR). This regression determines whether a category of emotion can reliably be predicted based on changes of the firing of a neuron relative to background and provides a single test for each neuron which determines both that the response is different from background firing and provides information distinguishing between different emotions. The results of MLR analyses of the total and left amygdala are described in Tables 5, 6. Notably, neurons in males were most highly responsive to stimuli with neutral affect, whereas neurons in females were most responsive to positive affect. Fisher's exact tests comparing the proportions of neurons affected by specific categories of emotion in men and women found a significant difference between genders in the total amygdala (*p* = 0.0054) and left amygdala (*p* = 0.0013).

**Table 5:** **Neurons significant for specific emotions relative to background firing in the total amydala**.

	**Female**	**Male**
Total # neurons	130	106
Sig. for positive	22	12
Sig. for negative	6	1
Sig. for neutral	3	13
Sig. for positive and negative	2	1
Sig. for positive and neutral	1	3
Sig. for negative and neutral	0	2
Sig. for all	1	0
Not sig. for any emotion	95	74
Fisher's exact test *p*-value	0.0054	

**Table 6:** **Neurons significant for specific emotions relative to background firing in left amygdala**.

	**Female**	**Male**
Total # neurons	76	66
Sig. for positive	18	11
Sig. for negative	6	0
Sig. for neutral	2	12
Sig. for positive and negative	1	1
Sig. for positive and neutral	1	1
Sig. for negative and neutral	0	2
Sig. for all	1	0
Not sig. for any emotion	47	37
Fisher's exact test *p*-value	0.0013	

As an additional test that neuronal responses to the three emotions differed from background firing, we tested whether the responses of neurons with a significant effect of emotion had responses which significantly differed from background firing, using the changes from background test (Steinmetz and Thorp, 2013). We found that 89% of neurons with a significant effect of emotion also had firing which differed significantly from background. Overall, the percentage of neurons whose responses differed significantly from background across all brain areas was 28%, which demonstrates a general effect of viewing an image of a face vs. the black background.

Finally, we note that men and women differed in performance of the manual emotion identification task. Consistent with the findings of prior studies (Hampson et al., 2006), we found that women identified the facial emotions of the stimulus images more quickly than men (see Figure S1). Additionally, males in the study correctly identified stimulus affect nearly 20% more often than females did (see Figure S2). We did not, however, find a significant correlation between behavior and neural responses on a trial by trial basis.

## Discussion

We found that neurons in the left amygdalae of men were more responsive to emotional faces than neurons in the same location in women. Logistic regression analyses demonstrated that neurons in men were most responsive to faces with neutral expressions, while those in women were most responsive to faces with positive expressions.

One prior publication reported human amygdalar neuron firing in response to the presentation of emotional face (Fried et al., 1997). Our results in Table 1 are in good agreement with these prior results as 10% of neurons in the amygdala overall in the present study responded to emotion. Our results differ from those reported by (Fried et al., 1997), that 10–20% of neurons in the amygdala responded to the emotion shown on a face (depending on mnemonic task) and that 10% of these neurons responded to the gender of the presented face, though they did not examine differences between genders of the subjects. Kawasaki et al. (2005) who found that 21% of neurons in the human ventromedial prefrontal cortex (VMPFC) responded selectively to the presentation of complex emotional scenes. Results in Table 1 show that 12% (9/174) of neurons in ventromedial prefrontal cortex respond selectively to the emotion shown on a face. One possible cause of this difference in the reported fraction of responsive neurons in the VMPFC is that Kawasaki et al. (2005) showed complex scenes designed to reliably signal strong and specific emotions (Lang et al., 2005), whereas our adventitous analysis was limited to faces depicting specific emotions.

Our experimental design required subjects to actively assess and identify the emotional state of presented faces. Completion of task trials therefore involved explicit emotional processing, as opposed to implicit processing, which pertains to the assessment of non-emotional criteria (e.g., gender or age). While inconsistencies within the neuroimaging literature have called into question whether the amygdala has greater activation under explicit or implicit experimental conditions (Habel et al., 2007), this work demonstrates unequivocally that the amygdala indeed plays an active role in emotional processing under explicit conditions.

Although previous work utilizing non-invasive techniques to assess gender differences in hemodynamic responses to emotional stimuli has produced conflicting results (Sergerie et al., 2008; Fusar-Poli et al., 2009; Stevens and Hamann, 2012), women are most frequently reported to be the more responsive gender in the neuroimaging literature. Heightened BOLD signals in women, particularly within the amygdala, have been reported to correlate with negative imagery (Klein et al., 2003; Domes et al., 2010; Frewen et al., 2011; Young et al., 2013); this finding is commonly concluded to contribute to higher rates of affective disorders in women (Ohrmann et al., 2010).

By using single-neuron recording to directly measure neural activity within the amygdala, we obtained results which suggest the opposite; namely, that neurons in amygdalae of men are more responsive to emotional stimuli than those in women. There are several possible explanations for the difference between our results and the bulk of those obtained via neuroimaging. Firstly, the relationship between the BOLD signal and underlying neural activity is still not well-understood (Sirotin and Das, 2009; Ekstrom, 2010; Boynton, 2011; Handwerker and Bandettini, 2011; Kleinfeld et al., 2011; Martin, 2014), and may well depend on the particular task being performed and the brain area involved (Sirotin and Das, 2009; Conner et al., 2011; Cardoso et al., 2012; Huo et al., 2014; Lima et al., 2014). Thus, the discrepancy between these results may simply reflect a different relationship between neural firing and the BOLD signal in the amygdala.

Secondly, discrepancies within the neuroimaging literature have been attributed, amongst other factors, to variations in experiment design (Derntl et al., 2012) and our behavioral task differed from those used in most prior studies, particularly regarding the nature of the stimuli. All emotional faces presented as stimuli were ostensibly male, designed originally to test neural response to race in a task in which gender was standardized. Since, viewer sex and sex of presented images have been shown to influence results of fMRI studies as well as behavioral identification tasks (Proverbio et al., 2012; Spreckelmeyer et al., 2013), it is possible that neural responses are largest for own-sex faces, as has also been shown an ERP study (Doi et al., 2010).

Both of these potential explanations suggest an interesting avenue for future research: how would single-neuron firing rates differ between men and women when viewing own-sex vs. opposite sex faces? If sample sizes permit, it may also prove intriguing to include additional subject variables which have been shown to influence BOLD signals in neuroimaging studies, including participant sexual preference (Perry et al., 2013) and menstrual cycle phase in female participants (Derntl et al., 2008). Since, women have been shown to respond significantly more quickly and accurately to human vs. avatar faces in an emotion identification task, with no comparable result observed in men (Moser et al., 2006), it would also be desireable to conduct this study using actual photographs showing emotional expressions rather than avatar faces.

Intriguingly in this study, males were more accurate in identifying the emotion expressed on faces whereas women had shorter response times. While these differences did not correlate with neural firing on a trial-by-trial basis, the overall changes in neural firing suggest that the higher accuracy of males may be related to larger changes in neural firing which are present in the left amygdala of male subjects. We did not observe a specific neural firing rate correlate of the faster responses in females.

Several limitations of this study should be noted. Firstly, the stimulus images in this study were designed to portray, as opposed to evoke, emotions. While these images were not designed to evoke emotion, it has been shown repeatedly that the viewing of emotional faces evokes the presented emotion in the participant, particularly with regards to “strong” emotions including happiness and sadness (Hess and Blairy, 2001; Wild et al., 2001).

Secondly, due to the invasive nature of single-unit recording, participants could not be chosen at random. All subjects shared a common diagnosis of refractory epilepsy, and were thus neurologically distinct from the majority of the population. We are not aware, however, of any reports that epileptics process emotion in a fashion distinct from that of the general public, nor that it has been demonstrated that men and women with epilepsy have gender-linked differences in emotional responses unequal with those observed in healthy individuals.

What is the potential cause of these gender based differences in neural responses to emotional faces? Given the limitations of this adventitous experimental design, we can speculate that these differences may reflect evolutionary differences accrued as a means of survival under respective selective pressures. Men have born the brunt of intergroup conflict dating back to hunter-gatherer societies (Van Vugt, 2009), and behavioral vestiges of this pattern have been consistently shown to persist to modern day. Cross-culturally, male-on-male violence accounts for more than half of homicide crimes (Jason et al., 1983; Eckhardt and Pridemore, 2009; Häkkänen-Nyholm et al., 2009), while the vast majority of homicides that do involve female victims or perpetrators take place between family members or acquaintances (Kellermann and Mercy, 1992). Extending to the modern day, males near-exclusively bear the brunt of violence from strangers or outgroup individuals. Men may have evolved greater responsiveness to potential social cues in male expressions due to their higher likelihood of encountering direct physical threat from other males in the evolutionary landscape (McDonald and Navarrete, 2012) (women, by contrast, would have been more likely to face sexual threat, the results of which would not necessarily impair reproductive fitness). This hypothesis is supported by our finding that males are most responsive to neutral faces. Also supporting this hypothesis, a 2009 study by Hareli et al. (2009) found that neutral expressions in men, but not in women, were perceived as being more dominant than men expressing sadness or happiness, and thus sensitivity to this expression would enable male viewers to better avoid physical altercation.

Alternatively, the observed results may be due to differences in inter- vs. intra-sex male expression of empathy, an emotion whose neuronal network has been shown in imaging studies to include the amygdala (Völlm et al., 2006). Prior work demonstrating that men are better able to correctly identify the expressions of male faces attributed this result in part to greater activation of the amygdala, specifically with regards to its role in empathy (Schiffer et al., 2013). While this may account for the heightened firing rate in this structure in males following the presentation of male stimuli, it fails to account for the lack of a similarly heightened rate in females, as the latter have been shown to be the more empathetic sex across a myriad of tests utilizing a wide range of techniques (Christov-Moore et al., 2014).

In summary, our findings demonstrate emotional differences between men and women at the single neuron level, thereby illustrating the profound effect of gender on the human brain. This finding necessitates the inclusion of subject gender as a potential variable in single-unit recording studies, particularly those whose scopes include firing rates within the amygdala. Gender should also be a factor of interest in single-unit experiments which utilize emotional imagery or human expressions.

## Author contributions

MN analyzed data and created text and figures. PS Designed and conducted experiments and created text and figures. KS Performed patient surgeries. DT supervised patient safety and clinical compliance.

### Conflict of interest statement

The authors declare that the research was conducted in the absence of any commercial or financial relationships that could be construed as a potential conflict of interest.

## SI methods

### Validation of designations of emotion

The intended emotion designations were validated in two perceptual experiments. Subjects were undergraduate volunteers from ASU with normal or corrected vision. (Epilepsy patients from the present study did not participate in validation testing.) In the first experiment (*n* = 25), subjects were shown all faces in random order and were asked to quickly classify each one as either “positive” or “negative” in expression, with no option to respond “neutral.” Variations in apparent race were included in the experiment but were orthogonal to the task. Positive faces were classified as positive in 93.2% of all trials, with a mean correct response time (RT) of 515 ms. Negative faces were classified as negative in 98.1% of all trials, with a mean correct RT of 499 ms. Neutral faces were classified as positive in 41.5% of trials, with a mean RT of 688 ms and as negative in the remaining 58.5% of trials, with a mean RT of 601 ms. This indicated that positive and negative expressions were quickly and accurately appreciated. Neutral faces generated more evenly divided responses, and all responses were far slower relative to non-ambiguous faces. There was, however, a bias toward interpreting neutral expressions as being slightly more negative.

Figure S1 **Tufte-style box plot (21) of mean first response time for each experiment by affect and gender.Response times are grouped by affect and gender**. The center dots show intragender means. Vertical lines extend from $\frac{\pm \left(1.58*IQR\right)}{\sqrt{\left(n\right)}}$ (where *IQR* = interquartile range, *n* = number of observations; equivalent to a 95% confidence interval for differences between medians, Chambers et al., 1983, p. 62) to the data point furthest from the median which is no more than ±(1.5^*^*IQR*)beyond the first or third quartiles. Both males and females were slowest to identify neutral expressions, and the largest gap in response time was in response to negative faces.

Figure S2 **Tufte-style box plot (21) of mean percentage correct of identification of affect of stimuli by experiment**. Percentage correct is grouped by gender. The center dots show intragender means. Vertical lines extend from $\frac{\pm \left(1.58*IQR\right)}{\sqrt{\left(n\right)}}$ (where *IQR* = interquartile range, *n* = number of observations; equivalent to a 95% confidence interval for differences between medians, Chambers et al., 1983, p. 62) to the data point furthest from the median which is no more than ±(1.5^*^*IQR*)beyond the first or third quartiles. Open circles show responses outside that range. Males had higher accuracy in the expression identification task than females across all categories of affect.
