# Supplementary figures and images for: Gender differences in human single neuron responses to male emotional faces
Source: Front Hum Neurosci. 2015 Sep 14;9:499. doi: 10.3389/fnhum.2015.00499 (PMC4568342; doi:10.3389/fnhum.2015.00499)

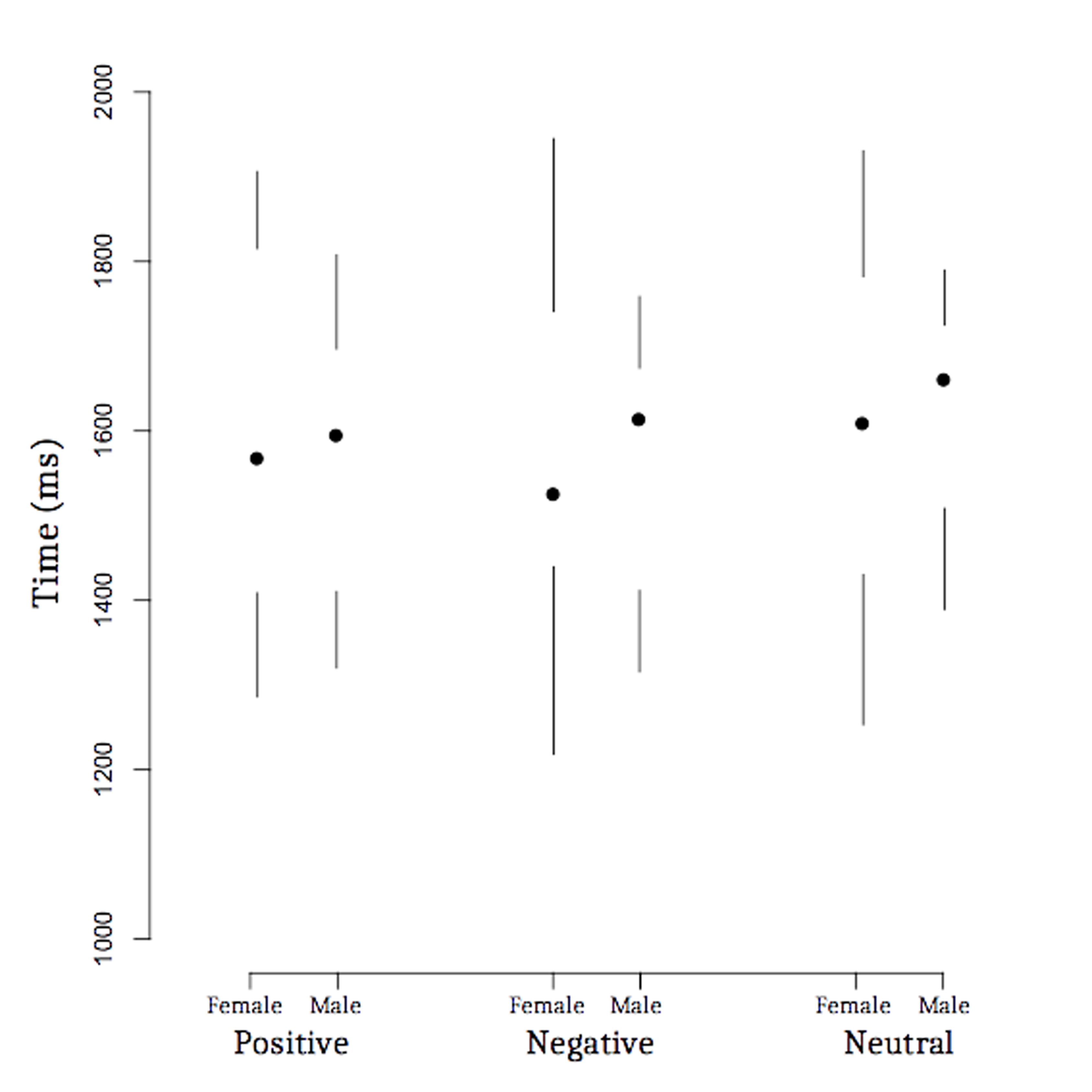

Supplement: Figure S1 — Tufte-style box plot (21) of mean first response time for each experiment by affect and gender.Response times are grouped by affect and gender. The center dots show intragender means. Vertical lines extend from ±(1.58*IQR)(n) (where IQR = interquartile range, n = number of observations; equivalent to a 95% confidence interval for differences between medians, Chambers et al., 1983, p. 62) to the data point furthest from the median which is no more than ±(1.5*IQR)beyond the first or third quartiles. Both males and females were slowest to identify neutral expressions, and the largest gap in response time was in response to negative faces. [file Image1.TIF]

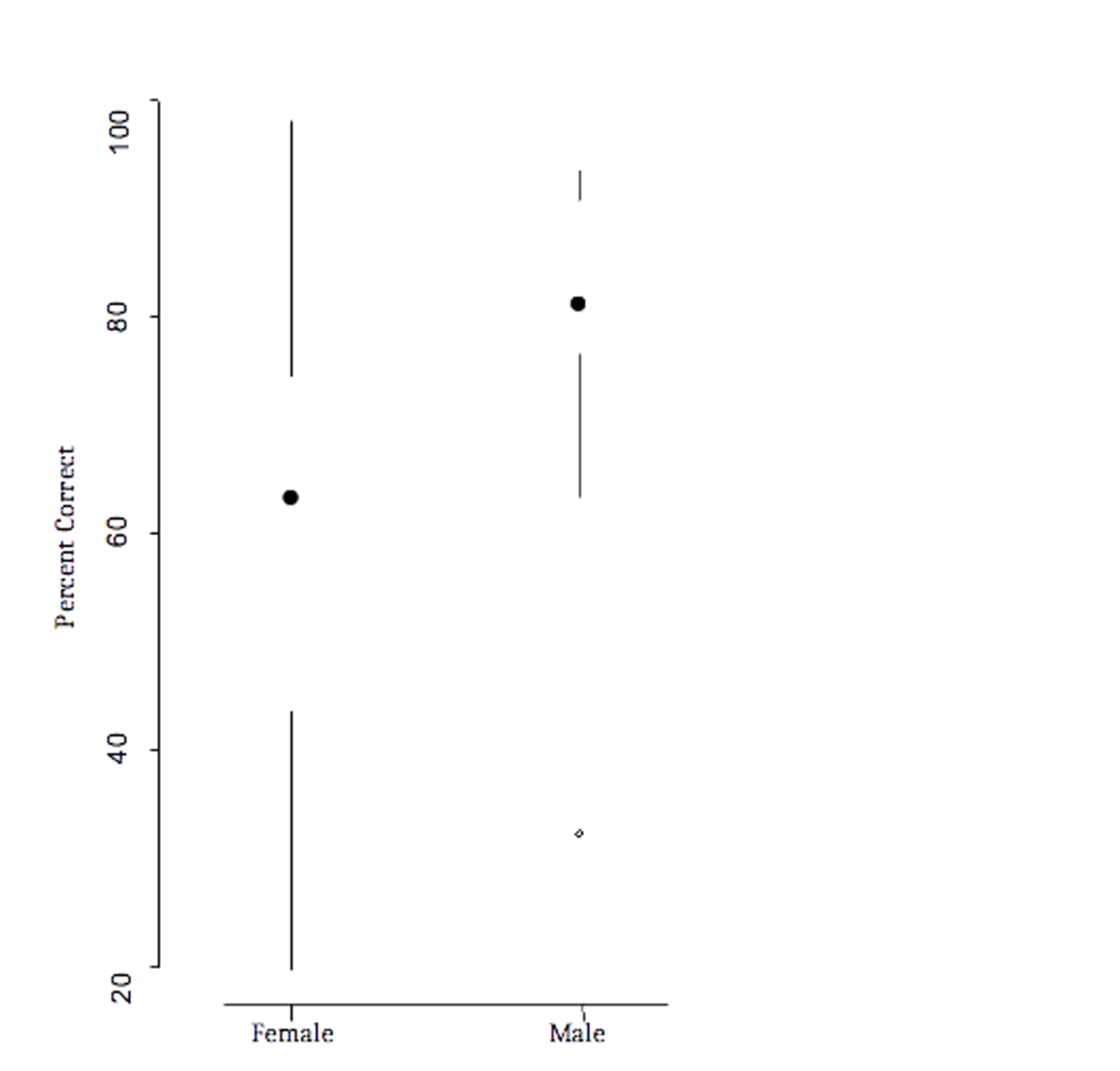

Supplement: Figure S2 — Tufte-style box plot (21) of mean percentage correct of identification of affect of stimuli by experiment. Percentage correct is grouped by gender. The center dots show intragender means. Vertical lines extend from ±(1.58*IQR)(n) (where IQR = interquartile range, n = number of observations; equivalent to a 95% confidence interval for differences between medians, Chambers et al., 1983, p. 62) to the data point furthest from the median which is no more than ±(1.5*IQR)beyond the first or third quartiles. Open circles show responses outside that range. Males had higher accuracy in the expression identification task than females across all categories of affect. [file Image2.TIF]
